# Predicting maize hybrid performance with machine learning and a locus-specific weighted degree of dominance transformation

**DOI:** 10.3389/fpls.2026.1694707

**Published:** 2026-04-01

**Authors:** Bright Enogieru Osatohanmwen, Indalécio Cunha Vieira, Mahmood Gholami, Cathy C. Westhues, Ahmad Reza Sharifi, Timothy M. Beissinger

**Affiliations:** 1 Division of Plant Breeding Methodology, Department of Crop Sciences, University of Goettingen, Goettingen, Germany; 2 Center for Integrated Breeding Research, University of Goettingen, Goettingen, Germany; 3 KWS SAAT SE & Co. KGaA, Einbeck, Germany; 4 Heritable Agriculture Inc., San Carlos, CA, United States; 5 Division of Animal Breeding and Genetics, Department of Animal Sciences, University of Goettingen, Goettingen, Germany

**Keywords:** dominance effects, genetic architecture, genomic prediction, gradient boosting, machine learning, maize, yield

## Abstract

The genetic architecture of a trait plays a vital role in the predictive ability of genomic models. While classical methods such as genomic best linear unbiased prediction (GBLUP) remain widely used in plant breeding, the value of machine learning (ML) is increasing because of its ability to capture non-linear effects. This study assessed ML and classical models incorporating a locus-specific weighted dominance effect transformation matrix for genomic prediction in hybrid maize. We evaluated five models in simulated and real hybrid maize dataset: (1) XGBoost with transformed SNP marker matrix (ML_Transformed), (2) XGBoost with conventional SNP marker matrix (ML), (3) GBLUP with additive effects only (AM), (4) GBLUP with additive and dominance effects (ADM), and (5) GBLUP with transformed SNP marker matrix (CADM). Two hybrid maize dataset were simulated, polygenic and oligogenic with dominance levels ranging from 0% to 40% while the real maize hybrid dataset evaluation consisted of traits with diverse genetic architectures, including grain yield, test weight, ear height, plant height, pollen and silk days after planting, and grain moisture. Results showed that the dominance transformation had mixed effects: it did not enhance ML performance, but only improved CADM in simulated scenarios. Across both simulated and real data, ML generally exceeded GBLUP performance, except in polygenic simulations where CADM outperformed all other models including the ML models. We also found that increasing dominance levels generally reduced predictive accuracy, regardless of the model. In general, these results suggest that CADM and ML_Transformed are promising for application in plant breeding. However, their success depends on the underlying traits genetic architecture, highlighting the importance of dominance-incorporating and trait-adaptable approaches to genomic prediction for optimizing breeding strategies.

## Introduction

Developments in molecular genetics technologies in the late 80s and early 90s led to the application of molecular markers for selection in plant and animal breeding. These markers were initially used primarily to detect Quantitative Trait Loci (QTL) and applied through Marker Assisted Selection (MAS) (Lande and Thompson, 1990; Collard & Mackill, 2008; Hasan et al., 2021). Although MAS is still used in breeding, the development of high-density genotyping platforms enabled the systematic detection and exploitation of abundant genomic variants particularly single-nucleotide polymorphisms (SNPs), which led to the development of genomic prediction models (Meuwissen et al., 2001). Genomic prediction involves building a model using the marker data spanning the entire genome, coupled with phenotypic data from sampled genotypes to predict future genomic breeding values (Lee et al., 2017; Tan et al., 2017).

The accuracy of predicted genotype performance is the primary measure of the effectiveness of a genomic prediction model, which is an essential tenet of genomic selection in plant and animal breeding. Factors that often affect the accuracy are the type and performance of the model, individual relatedness, marker density, sample size, genetic architecture, and heritability of the trait (Hayes and Goddard, 2010; Ober et al., 2012). An essential factor, genetic architecture, refers to the rules governing how multi-locus genotypes contribute to phenotypic variation and how different factors influence this contribution. Possible factors that influence the contribution include the number of loci and their genetic position, the number of alleles per locus, the level of individual contribution of a single locus, pleiotropy patterns, and the mode of gene action, which can be additive, dominant, or epistatic (Fu et al., 2013). Additive effects usually explain a large portion of genetic variance.

Nevertheless, dominance effects (non-additive) have also been shown to make contributions to genetic variance, and several previous efforts have been devoted to modeling dominance in genomic prediction models (Vitezica et al., 2013; Amadeu et al., 2020; Bajgain et al., 2020; Ramstein et al., 2020; González-Diéguez et al., 2021; Tan and Ingvarsson, 2022). These studies presented statistical methods for adding dominance genetic effects into genomic prediction, highlighting both the advantages and disadvantages of using dominance effect estimates. To further the efforts of including dominance effects in classical genomic prediction, a recent method Genomic BLUP model combining additive and dominance genetic effects (CADM) was introduced by (Liu et al., 2022), which integrates additive and dominance effects through locus-specific weights on heterozygous genotypes, and demonstrated an improved predictive accuracy in livestock species (Poultry and Pig) particularly for traits with high broad sense heritability.

Despite significant advances in classical genomic methods that incorporate non-additive genetic effects, the prediction of complex traits in breeding remains a challenging task and is open to improvements.

Machine learning (ML) is a branch of artificial intelligence that enables computers to learn from data without being explicitly programmed, encompassing a class of computational methods capable of identifying complex patterns and relationships with minimal prior assumptions about the underlying structure. By reducing the need for manual feature engineering and explicit model specification, ML substantially lowers the human effort required to extract information from large and complex datasets (Chafai et al., 2023; Cheng & Wang, 2024). Unlike classical parametric models, ML approaches can capture nonlinear effects and high-order interactions among predictors. In plant breeding, the increasing availability of high-dimensional genomic, phenotypic, and environmental data has positioned ML as a powerful complement to traditional quantitative genetic methods. ML techniques have shown promise for improving the prediction of complex traits, hybrid performance, and genotype-by-environment interactions, thereby supporting more efficient selection decisions in modern breeding programs (Crossa et al., 2017; Montesinos-López et al., 2018; de los Campos et al., 2013; Azodi et al., 2019). The use of machine-learning methods has increased over the years, and there have been reports of improved genomic accuracy for the prediction of phenotypic traits with these methods (Jeong et al., 2020; Montesinos-López et al., 2022; Zhao et al., 2020; Sousa et al., 2020; Sirsat et al., 2022; Mora-Poblete et al., 2023). An increase in the prediction accuracy of machine learning models has been reported to result from the models’ ability to capture non-linear relationships between genome and phenotype (Sousa et al., 2020; Sirsat et al., 2022; Costa et al., 2022). Even though machine learning methods have the potential to capture non-linear relationships, there have been some instances where model adjustments have enhanced their performance. Some of such adjustments were captured in research by Budhlakoti et al. (2022) and Zhao et al. (2021), who both investigated the fusion of classical and ML techniques to improve the performance of machine-learning models in genomic prediction.

Budhlakoti et al. (2022) proposed an integrated estimator combining genomic best linear unbiased prediction (GBLUP) and Support Vector Machine (SVM) predictions. This hybrid model weights the contributions of both methods based on their error variances, thereby improving the model’s ability to use both additive and non-additive effects. The approach was evaluated using simulation data and found to improve predictive ability and reduce error variance compared to standalone models. Zhao et al. (2021) presented a method named NN-Bayes in which the framework combines SNP data via Bayesian alphabet models with unobserved intermediate characteristics (hidden nodes). It uses a non-linear activation function to model relationships between hidden nodes and observed traits. This hybrid approach accommodates both additive and non-additive genetic effects. It uses Markov Chain Monte Carlo (MCMC) methods to ensure accurate posterior distribution inference, which can be used for significance testing and association studies. By integrating the strengths of both approaches (classical and ML methods), these studies have aimed to achieve a more robust and versatile predictive framework capable of more effectively capturing complex allelic interactions. Both studies reported fluctuating performances with simulated and real data across different species. In a parallel line of research, Mathew et al. (2022) explored data transformation as a means to extract more meaningful information for genomic prediction. They introduce a new method called NeuralLasso for genomic prediction, inspired by neural networks and incorporating traditional elements of LASSO. This approach addresses the challenge that neural networks require large datasets for effective learning due to their multiple layers. With the tested cases, they reported an improved prediction accuracy over conventional methods, attributing this improvement to two main factors: first, NeuralLasso accounts for additive and higher-order local epistatic genetic effects, unlike conventional methods that typically consider two-loci genome-wide interactions, which may lose local epistatic effects due to recombination. Secondly, NeuralLasso can also consider other context-specific effects beyond epistasis, as demonstrated by its high predictive performance even in scenarios with expected minimal or no epistasis.

Though outcomes vary, the above approaches aim for more robust predictive frameworks, with some studies showing notable improvements while others face challenges. In this study, we propose a machine learning method that incorporates the locus-specific degree of dominance transformation used in the CADM model as a prior for genomic prediction in hybrid maize. The ML method used here was extreme gradient boosting machine (XGBoost). While XGBoost have been used in genomic prediction and related classification and regression tasks, their direct integration with dominance parameterization for genomic prediction remains unexplored. To the best of our knowledge, this is the first study to combine dominance information (from CADM) and ML model for hybrid maize performance prediction. Unlike conventional ML approaches that treat marker data as generic features, our method preserves quantitative genetic structure by explicitly encoding dominance effects prior to model fitting, thereby bridging classical genetic theory with modern ensemble learning.

Our study has two primary objectives:

1. To evaluate the predictive performance of the CADM method using both real and simulated hybrid maize data, where the simulated datasets were designed with controlled genetic architectures to systematically assess model behavior across varying dominance variance levels and trait heritability.
2. To investigate the performance of the extreme gradient boosting machine (XGBoost) combined with the locus-specific degree of dominance transformed SNP marker matrix (as in CADM) for genomic prediction in real and simulated hybrid maize data under similar controlled conditions.

We benchmark the CADM model against two classical GBLUP alternatives: (i) a GBLUP with additive effects only (AM), and (ii) a GBLUP with additive and dominance effects (ADM). Furthermore, we compare CADM with two machine learning approaches: (i) XGBoost with the conventional SNP marker matrix (ML), and (ii) XGBoost with locus-specific weighted dominance effects transformed SNP marker matrix (ML_Transformed). These comparisons enable a comprehensive assessment of the utility of locus-specific dominance modeling in both classical statistical approaches and machine learning frameworks for genomic prediction in hybrid crops.

## Materials and methods

### Data simulation

**Figure 1:**
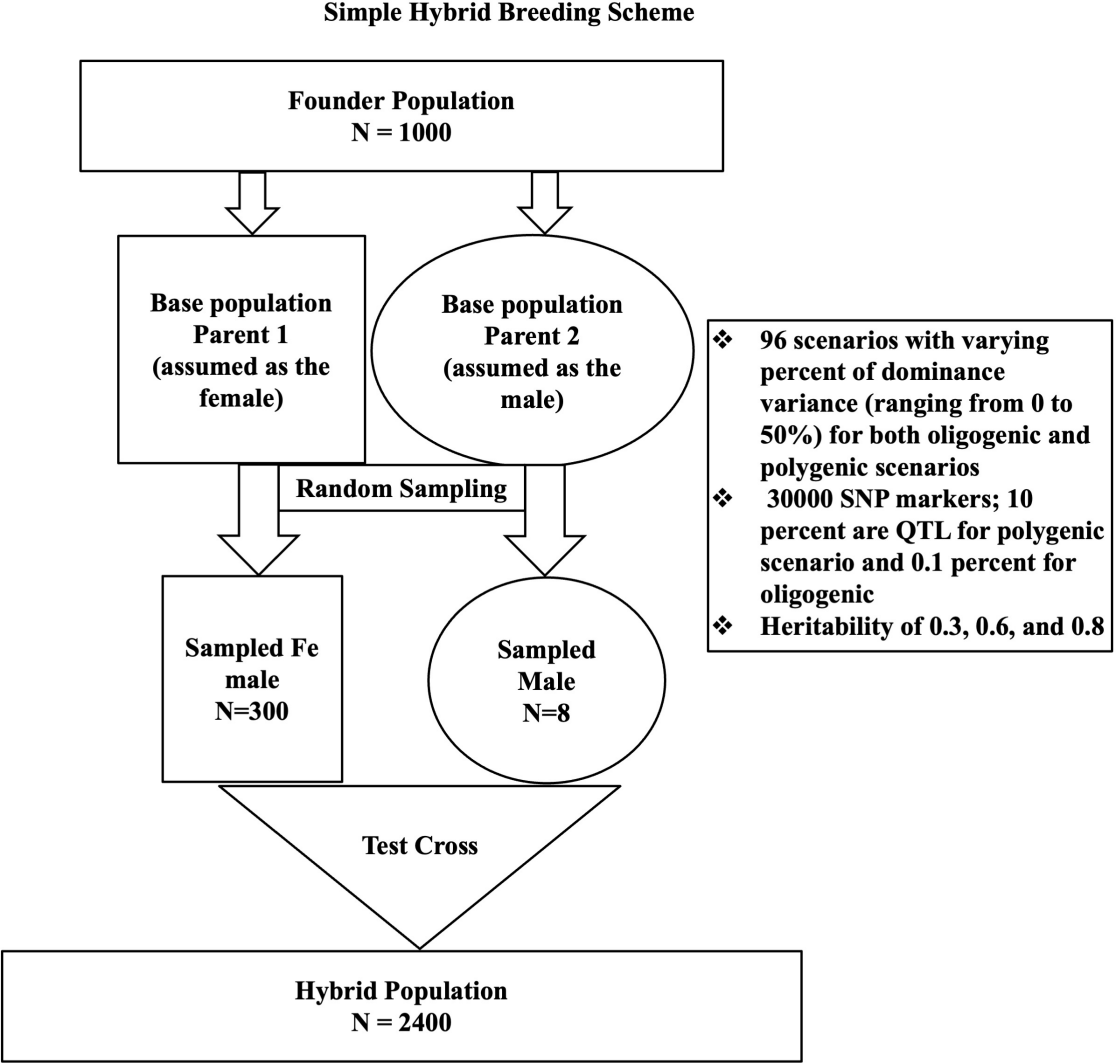
A simple hybrid breeding scheme showing the details of the simulated population. The first step was creating a Founder population of 1000 individuals. From this Founder population, two base populations were created that served as a genetic pool for Parent 1 (Female) and Parent 2 (Male). To get the individual used for the test cross, there was random sampling from both parental pools (300 females from Parent 1 and 8 males from Parent 2). The final step was a cross of both male and female sampled individuals.

To assess the combined model’s performance under varying dominance levels and trait architectures, a stochastic simulation of simple maize hybrid breeding was conducted using AlphaSimR (Gaynor et al., 2021). **Figure 1** shows the breeding scheme. We simulated 96 traits with different gene actions (the proportion of dominance ranged from 0 to 40%) at various levels of broad sense heritability (high heritability of 0.8, medium at 0.6, and low at 0.3). We assumed 30000 SNP markers and 300 QTLs on each of the ten chromosomes for the polygenic scenario and 30000 SNP markers and 3 QTLs on each chromosome for the oligogenic scenario. A total of 2400 hybrid individuals was simulated from a founder population of 1000 individuals (randomly taking 300 females and eight males from the founder population).

### Real data set

We use data from Genomes to Fields (G2F) 2022 Maize Genotype by Environment Prediction Competition (Lima et al., 2023). We used data across 4 years, from 2018 to 2021, which consisted of 2464 unique maize (*Zea mays L*.) hybrids evaluated in multiple environments across the United States of America, Canada, and Germany. The modified Randomized Complete Block Design (RCBD), mainly with two replications per environment, was used in the trials. Our analysis covers seven traits: Grain Yield (Mg ha^−1^), Test Weight (kg m^−3^), Ear Height (cm), Plant Height (cm), Pollen DAP (days), Silk DAP (days), and Grain Moisture (Days after pollination, DAP). The genotypic data were described in (Lima et al., 2023). For the G2F materials from 2014 to 2023, variant calls were performed using the Practical Haplotype Graph (PHG) (Bradbury et al., 2022). Hybrid genotypes were generated by combining information about their parent lines using the CreateHybridGenotypes plugin available in TASSEL 5 (Bradbury et al., 2007), yielding 4,928 individuals with 437,214 markers. We filtered for years 2018, 2019, 2020, and 2021 and excluded SNP markers with MAF of less than 0.05 and missingness of 0.2, resulting in 2464 unique hybrids and 217,287 SNP markers.

To identify and remove outliers, a linear model was fitted with hybrid and replicate as fixed effects in each unique environment, defined by field location and year. The model used is expressed as:


$$y_{ij}=\mu \;+H_{i}+R_{j}+e_{ij}\;\;$$

where $y_{ij}$ is the observed phenotypic value of the *i*-th hybrid of the *j*-th replicate; μ is the overall mean; $H_{i}$ is the fixed effect of the *i*-th hybrid; $R_{j}$ is the fixed effect of the *j*-th replicate $e_{ij}$ is the residual term associated with the experimental unit.

Residuals ( $e_{ij}$) greater than two standard deviations (σ) were removed:


$$|e_{ij}|>2\text{σ}$$

A two-step analysis was used to calculate the best linear unbiased estimates (BLUEs) for each hybrid. This was done to reduce computational time. In the first step, BLUEs with hybrid and replicate as fixed effects were conducted across the four years within each field location. The model used was the same one used for the outlier identification and removal. In the second step, a linear mixed model was used with hybrid as a fixed effect and environment (field location × year) as a random effect:


$$y_{ik}=\mu \;+H_{i}+E_{k}+e_{ik}\;$$

where $y_{ik}$ is the BLUE of the *i*-th hybrid calculated in the first step; μ is the overall mean; $H_{i}$ is the fixed effect of the *i*-th hybrid; $E_{k}$ is the random effect of the *k*-th environment; $e_{ik}$ is the residual term associated with the observation $\;y_{ik}$ The estimated values (BLUEs) obtained here were used for all subsequent analyses in this study.

### Variance components and heritability estimation

To create the additive (VanRaden, 2008) and dominance (Vitezica et al., 2013) relationship matrices used in the GBLUP method, we used the package AGHMatrix (Amadeu et al., 2016) from R 4.2.2 (R Core Team, 2022). The genetic variance of the traits in the simulated data was extracted from the simulation results from AlphaSimR (Gaynor et al., 2021). In contrast, the variance components of the real data set were calculated using emmreml and emmremlMultiKernel functions of the EMMREML package in R (Akdemir and Okeke, 2015). The emmremlMultiKernel function is a wrapper for the emmreml function that handles more than one component with known covariance structures. In contrast, the emmreml function uses the EMMA algorithm to solve a mixed model with a single known covariance structure (Kang et al., 2008). Like the genomic relationship matrices used in this study.

The proportion of dominance variation was calculated as the dominance variance divided by the total genotypic variance:


$$PDV=\frac{V_{d}}{V_{g}}$$

where $V_{g}=V_{a}+V_{d}\;$ represents the total genotypic variance, with $V_{a}$ and $V_{d}$ denoting additive and dominance variance components, respectively.

And another was calculated with respect to all phenotypic variance, denoted as:


$$d^{2}=\frac{V_{d}}{V_{p}}$$

where $V_{p}=V_{a}+V_{d}+V_{e}$ denotes the total phenotypic variance and $V_{e}$ represents the residual variance.

Narrow-sense heritability was calculated as the additive variance divided by the phenotypic variance:


$$h^{2}=\frac{V_{a}}{V_{p}}$$

And broad-sense heritability was calculated as the sum of additive and dominance variance divided by the total phenotypic variance:


$$H^{2}=\frac{V_{a}+V_{d}}{V_{p}}$$

### Genomic prediction models

#### Classical models

Three classical models were chosen for this study. These are GBLUP with additive effects only (AM), which uses a single genomic relationship matrix for additive effects (VanRaden, 2008), GBLUP with additive and dominance effects (ADM), which uses a genetic relationship matrix for both the additive and dominance effects using separate kernels for each (Vitezica et al., 2013), and GBLUP with locus-specific weighted dominance effects transformed SNP marker matrix (CADM), which combines the additive and dominance effects in a single genomic relationship matrix (Liu et al., 2022). These classical models have been extensively described by Liu et al. (2022). These models were selected to represent increasing levels of genetic complexity and mirror the machine learning equivalent in classical quantitative genetic methods. AM serves as a baseline widely used in genomic selection due to its simplicity and robustness. ADM extends this framework by explicitly modeling dominance effects, which are known to contribute to hybrid performance. CADM further integrates additive and locus-specific dominance information within a single genomic relationship matrix, enabling a more compact representation of non-additive genetic variation. CADM was also selected to provide direct comparison with its ML counterpart, it uses the same transformed matrix as one of the ML models used in this study. Together, these models provide a systematic benchmark for evaluating the contribution of dominance effects and for comparing classical quantitative genetic approaches with ML models.

#### Machine learning models

The machine learning models used in this study were fitted using XGBoost (Chen and Guestrin, 2016). It is an implementation based on a gradient-boosting Decision Tree (GBDT) but optimized by the highly efficient distribution of computing (Chen and Guestrin, 2016). The GBDT uses a technique that creates an ensemble of weak learners (decision trees), optimizing a chosen loss function at each boosting step. Then, new trees are fitted to the residuals of the previous one (Friedman, 2001).

**Figure 2:**
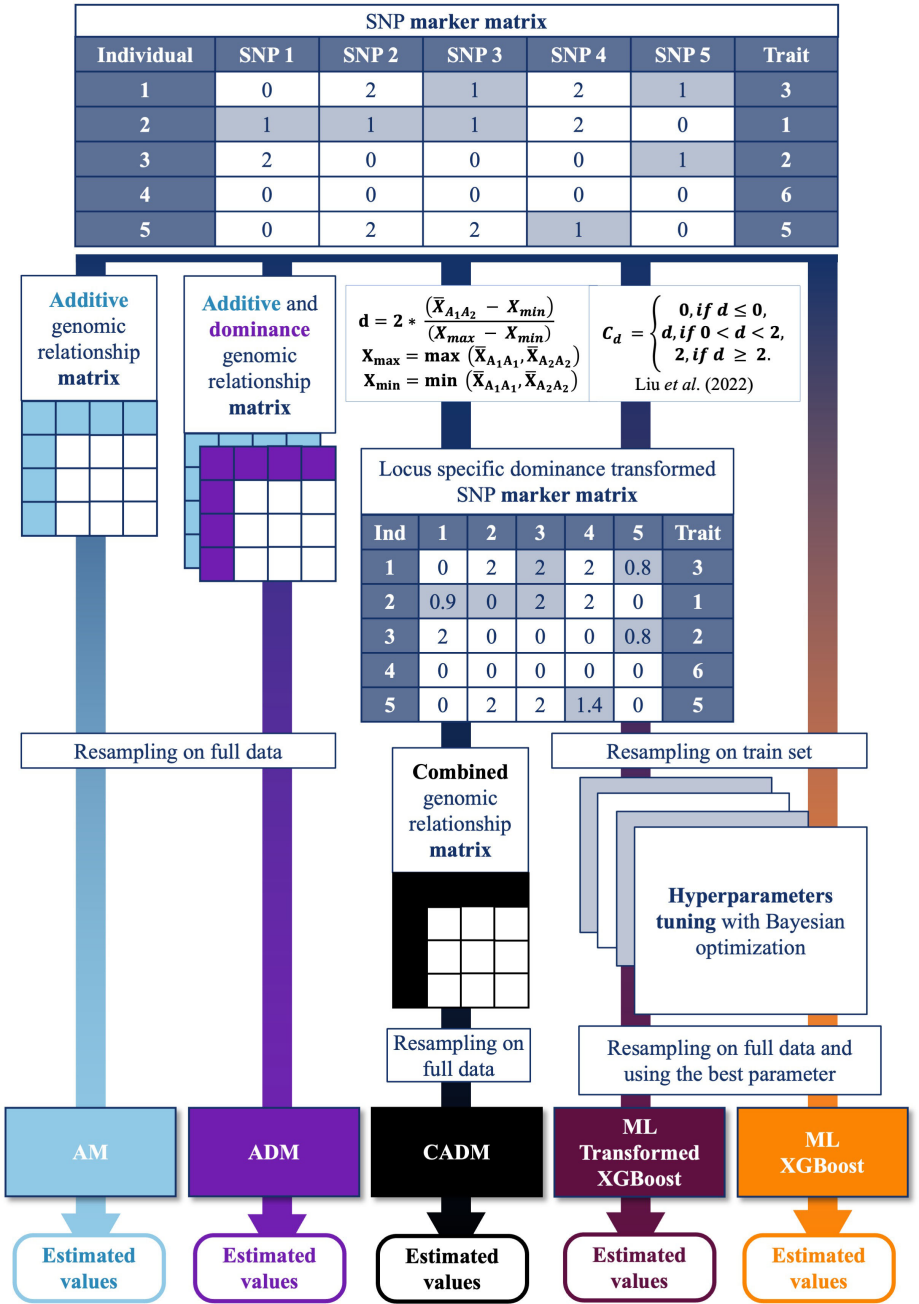
Workflow of genomic prediction models incorporating additive and dominance effects using classical method and a genomic prediction scheme of the locus-specific degree of dominance transformation steps using machine learning and classical method. The process starts with a SNP marker matrix, where individuals are genotyped across multiple SNP loci, and their corresponding trait values are recorded. The workflow produces estimated trait values from five models, including GBLUP Additive genetic effect model (AM), GBLUP Additive & Dominance effects model (ADM), Combined Additive & Dominance Model (CADM), ML Transformed (XGBoost), and ML (XGBoost). Three Models (AM, ADM, and ML) used the SNP marker. In contrast, for the other two (ML Transformed & CADM), a locus-specific weighted dominance transformation is applied to the SNP marker matrix, generating transformed values based on dominance degree calculations (CADM was created by Liu et al. (2022), and they extensively described CADM, ADM, and AM). This transformation results in a modified SNP marker matrix capturing locus-specific weighted dominance effects. For classical genomic prediction, GBLUP models are applied using the additive, additive + dominance, and transformed genomic relationship matrices, with model performance assessed through resampling on the full dataset. There is the additive genomic relationship matrix, which captures additive genetic effects; the dominance genomic relationship matrix, incorporating dominance effects; and the combined genomic relationship matrix, which uses the locus-specific dominance-transformed SNP marker matrix to incorporate both additive and dominance effects. For the machine learning approach (XGBoost), the data undergoes resampling on a training set, followed by Bayesian optimization for hyperparameter tuning. The optimized parameters are then applied to the resampled data from the complete dataset for final model evaluation.

Two ML models were fitted with the XGBoost method: the XGBoost conventional model (ML) that used the conventional SNP MARKER MATRIX, and the XGBoost combined model (ML_Transformed) that used the Transformed SNP MARKER MATRIX for the XGBoost prediction. See **Figure 2**.

The transformed marker SNP matrix was based on the assumption that the degree of dominance differs from one locus to another. The transformation is done by first calculating the degree of dominance based on the heterozygous and homozygous marker genotype deviation. The degree of dominance (**d)** is calculated described by Liu et al. (2022):


$$d=2*\frac{\left({\bar{x}}_{A_{1}A_{2}}-\;x_{min}\right)}{\left(x_{max}-\;x_{min}\right)}$$

where $x_{min}=min({\bar{x}}_{A_{1}A_{1}},\;{\bar{x}}_{A_{2}A_{2}})$, $x_{max}=max({\bar{x}}_{A_{1}A_{1}},\;{\bar{x}}_{A_{2}A_{2}})$ and ${\bar{x}}_{A_{1}A_{1}}$, ${\bar{x}}_{A_{1}A_{2}}$ and ${\bar{x}}_{A_{2}A_{2}}$ are the corrected phenotypes mean of the three genotypes $A_{1}A_{1}$, $A_{1}A_{2}$, and $A_{2}A_{2}$, respectively, with the assumption that $A_{1}$ homozygous ( $A_{1}A_{1}$) is coded 0, heterozygous ( $A_{1}A_{2}$) 1, and $A_{2}$ homozygous ( $A_{2}A_{2}$) 2. Then, the estimated degree of dominance (**d**) is used to construct the new marker matrix, replacing all heterozygous ( $A_{1}A_{2}$) sites of a locus with d for each locus. To avoid data leakage issues, **d** is calculated using the training set. See Liu et al. (2022) for more details.

#### Pre-processing of data and hyperparameters optimization

Hyperparameter tuning is an integral part of the ML process. The default hyperparameter does not always give optimal performance (Xiao et al., 2020; Vincent and Jidesh, 2023; Arnold et al., 2024). There are numerous approaches to hyperparameter optimization. This work used Bayesian optimization (utilizing an iterative Gaussian process) to choose the best hyperparameter.

Bayesian optimization is an efficient model-based optimization approach that iteratively searches the hyperparameter space by balancing exploration and exploitation. Bayesian optimization constructs a probabilistic surrogate model of the objective function, defined here as predictive performance under cross-validation, and uses an acquisition function to select new hyperparameter configurations that maximize expected improvement. By considering prior evaluations using different hyperparameter combinations at each iteration, the method focuses the search on regions of the parameter space associated with higher validation scores, while explicitly accounting for uncertainty in unexplored regions. This approach enables the identification of near-optimal hyperparameters with substantially fewer evaluations than exhaustive grid or random search strategies (Williams & Rasmussen, 2006; Snoek et al., 2012; Shahriari et al., 2015). This method was selected because it has been reported to be faster and more flexible than other approaches (Westhues et al., 2021; Chowdhury et al., 2022). For the Bayesian hyperparameter optimization, we used 20 iterations, three-fold cross-validation (CV), and mean squared error for scoring. The tuning was done on the training set only (which was further divided into train and validation sets for CV). See **Supplementary Table 4** for more info on the hyperparameter tuning. The best estimators from the Bayesian Hyperparameter tuning are then used for the whole data training and testing for each CV.

#### Model performance and cross-validation scheme

The GBLUP and ML models results were assessed and compared using the Pearson correlation coefficient (R) between predicted and observed values. A five-fold CV with ten repeats was used to estimate the accuracy of genomic prediction, and the results were averaged (for real data). All ML analyses were implemented in Python 3.8 with packages including XGBoost (Chen and Guestrin, 2016), scikit-learn (Pedregosa et al., 2018), and scikit-optimize (Head et al., 2021).

## Results

### Variance components and heritability estimates

**Supplementary Tables 1, 2** and **Supplementary Information** summarize variance components, the proportion of dominance variance (PDV), and heritability for polygenic and oligogenic simulated populations. Estimates from AlphaSimR and ADM model were highly consistent, with only minor discrepancies; AlphaSimR estimates were therefore used for visualization and downstream analyses.

PDV varies across simulated scenarios. In polygenic scenarios, PDV ranged from 0–45%, while in oligogenic scenarios it ranged from 0–55%. Corresponding $d^{2}\;$ ranged from 0–35% and 0–40%, respectively. Broad-sense heritability (H^2^) spanned approximately 30–80%, and narrow-sense heritability (h^2^) ranged from 15–80% across both genetic architectures.

**Figure 3:**
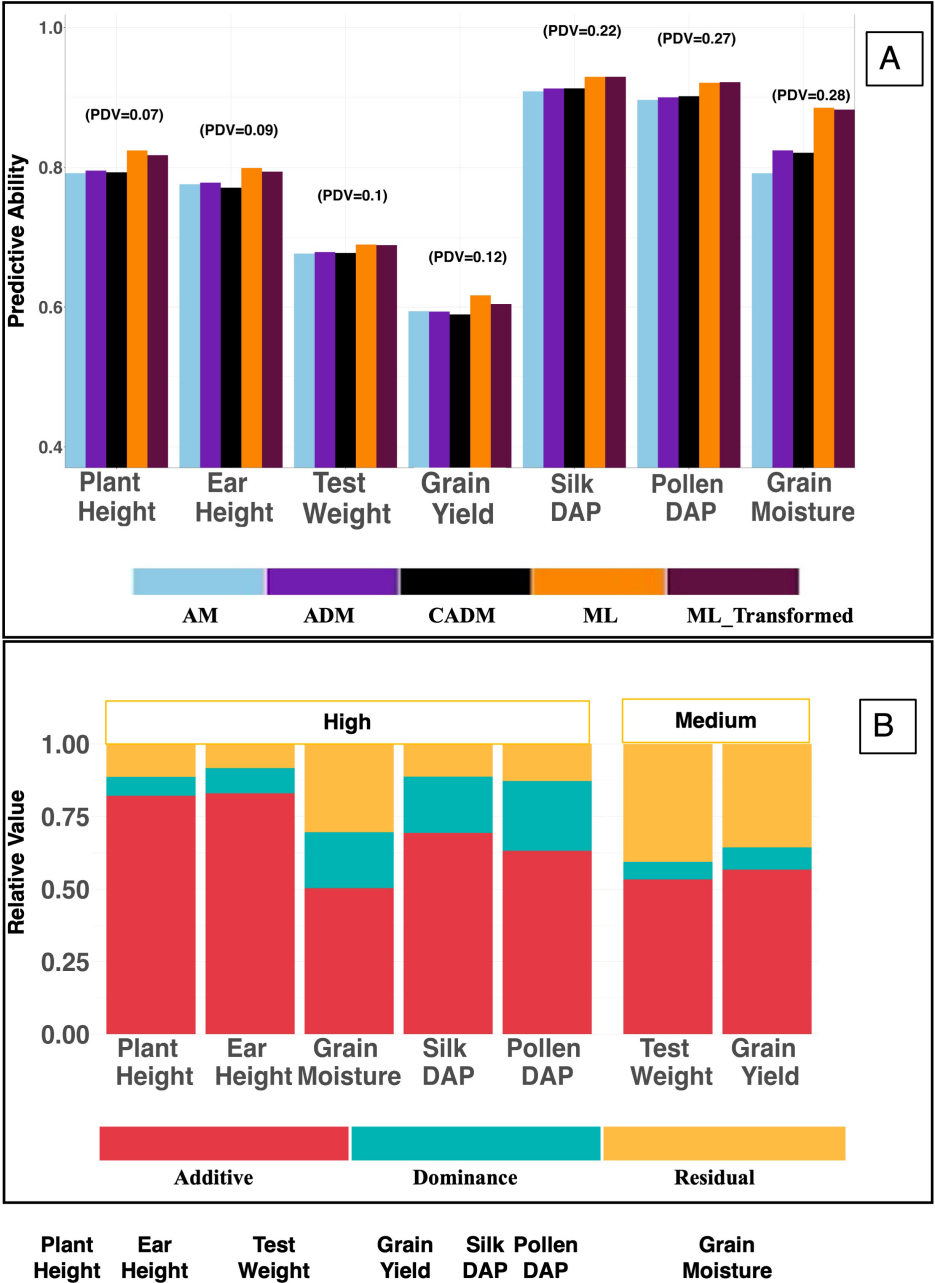
**(A)** Displays the predictive ability of all seven analyzed traits in the G2F population across the genomic models used in this study. The plot also captures the Proportion of Dominance Variance (PDV) for each trait. **(B)** Illustrates the relative values of variance components for all seven traits and categorizes each trait based on broad-sense heritability: High (≥0.7) or Medium (0.40–0.69). PDV, proportion of dominance variation is calculated as the ratio of dominance variance to total genotypic variance $PDV=V_{d}/V_{g}$, where $\;V_{g}=V_{a}+V_{d}$ represents the total genotypic variance, with $V_{a}$ and $V_{d}$ denoting additive and dominance variance components, respectively; $d^{2}\;,\text{ t}$he proportion of dominance variation, calculated as the dominance variance divided by the total phenotypic variance $d^{2}=V_{d}/V_{p}\text{, where }V_{p}=V_{a}+V_{d}+V_{e}\;$denotes the total phenotypic variance and $V_{e}\;$represents the residual variance. AM, GBLUP with additive effects only, using an additive genomic relationship; ADM, GBLUP with additive and dominance effects, using separate additive and dominance relationship matrices; ML, conventional XGBoost model using the original SNP marker matrix; ML_Transformed, XGBoost model using the transformed SNP marker matrix incorporating additive and dominance information.

In the real population (**Supplementary Table 3**; **Figure 3A**), PDV ranged from 7–28%, with $d^{2}\;$ between 6–24%. Although all traits exhibited dominance variation, grain yield, plant height, and test weight showed relatively low dominance contributions.

### Impact of Dominance Variation on Genomic Prediction Accuracy

**Figure 4:**
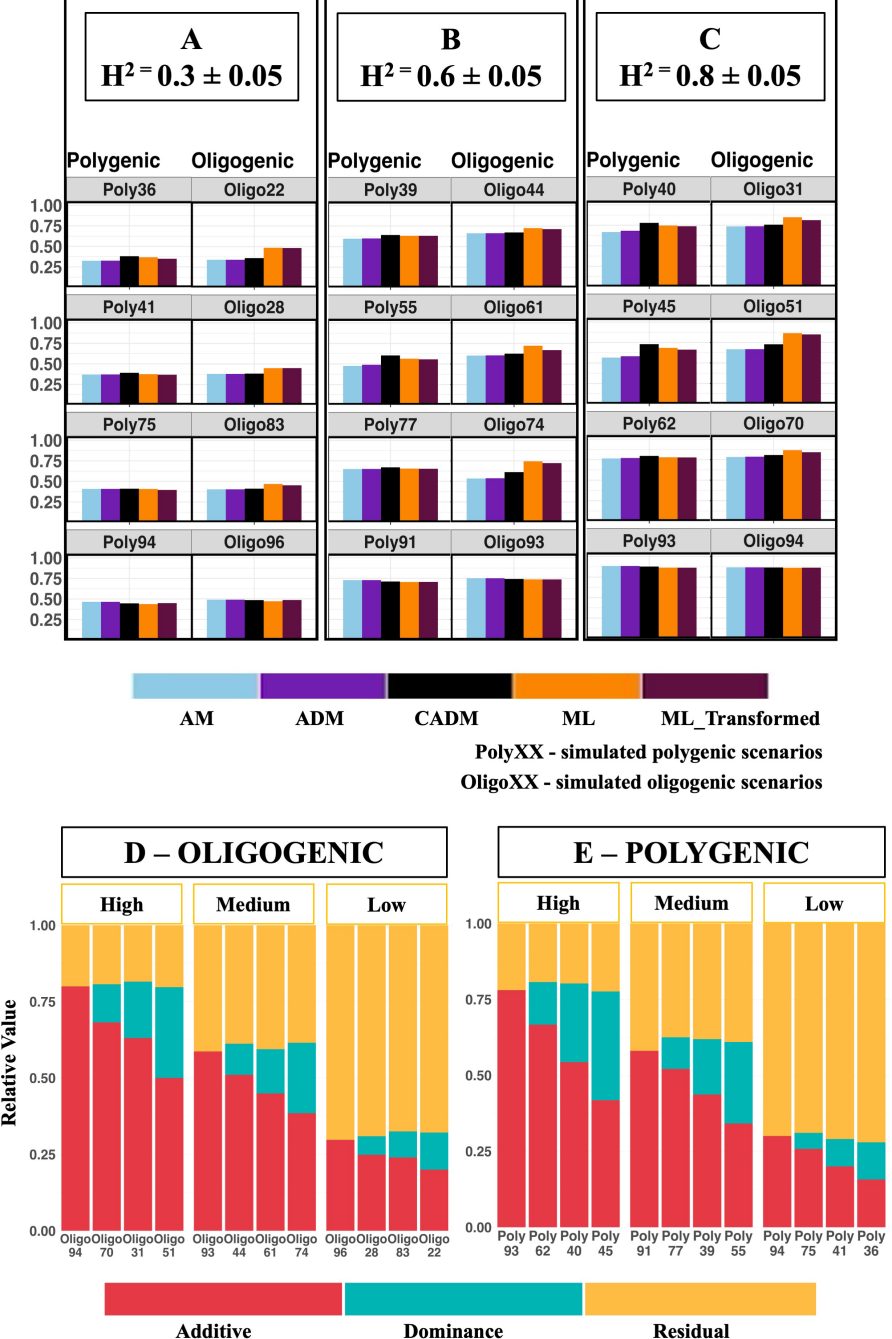
Shows the predictive ability (Pearson’s correlation between the observed and predicted) ofthe five models across the different proportion of dominance variance and the three levels of broad sense heritability for simulated oligogenic and polygenic scenarios (using selected scenarios to represent the different genetic architecture simulated). **(A)** Broad sense heritability of 0.3 ± 0.05 **(B)** Broad sense heritability of 0.6 ± 0.05 **(C)** Broad sense heritability of 0.8 ± 0.05. See **Supplementary Table 1, S2** for d^2^ and the PDV of each simulated scenario. (D&E) Relative values of variance components for simulated oligogenic **(D)** and polygenic **(E)** scenarios (using selected scenarios to represent the different genetic architecture simulated). PDV, proportion of dominance variation is calculated as the ratio of dominance variance to total genotypic variance $PDV=V_{d}/V_{g}$, where $\;V_{g}=V_{a}+V_{d}$ represents the total genotypic variance, with $V_{a}$ and $V_{d}$ denoting additive and dominance variance components, respectively; $d^{2}\;,\text{ t}$he proportion of dominance variation, calculated as the dominance variance divided by the total phenotypic variance $d^{2}=V_{d}/V_{p}\text{, where }V_{p}=V_{a}+V_{d}+V_{e}\;$ denotes the total phenotypic variance and $V_{e}\;$ represents the residual variance. All simulated polygenic genetic architecture scenarios are represented in the figure by “Poly” followed by a number (e.g., *Poly36*), with each label denoting a distinct polygenic scenario characterized by 3,000 loci with small-effect contributions. In contrast, all simulated oligogenic genetic architecture scenarios are represented by “Oligo” followed by a number (e.g., *Oligo22*), with each label denoting a distinct oligogenic scenario characterized by 30 loci with moderate-to-large effects.

**Figure 5:**
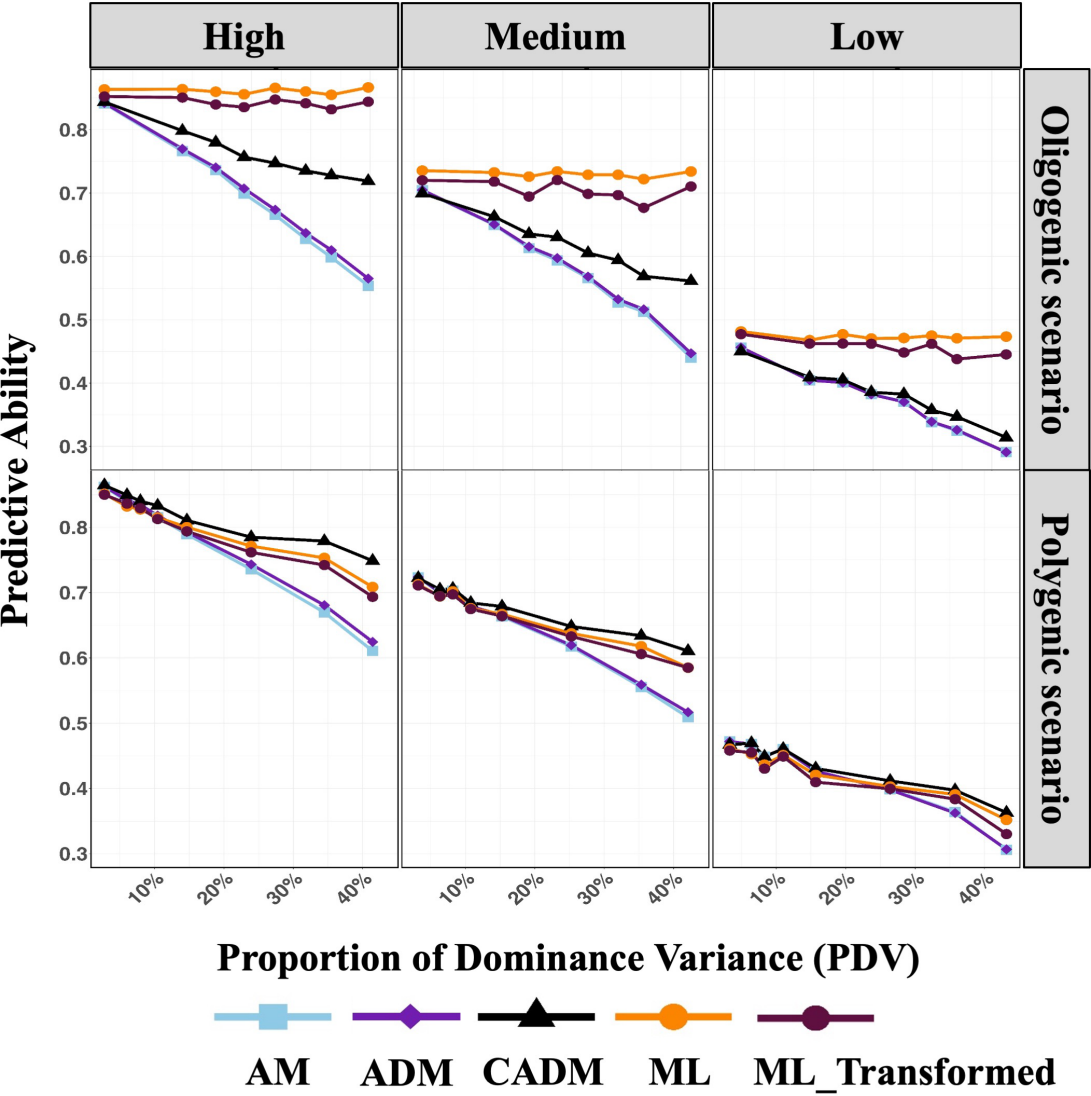
The average performance of the models using PDV from 0 to 50 percent, under three levels of broad sense heritability: High (0.7 and above), Medium (0.40-0.69), and Low (0.39 and below) for oligogenic and polygenic simulated scenarios. PDV, proportion of dominance variation is calculated as the ratio of dominance variance to total genotypic variance $PDV=V_{d}/V_{g}$, where $\;V_{g}=V_{a}+V_{d}$ represents the total genotypic variance, with $V_{a}$ and $V_{d}$ denoting additive and dominance variance components, respectively; $d^{2}\;,\text{ t}$he proportion of dominance variation, calculated as the dominance variance divided by the total phenotypic variance $d^{2}=V_{d}/V_{p}\text{, where }V_{p}=V_{a}+V_{d}+V_{e}\;$ denotes the total phenotypic variance and $V_{e}\;$ represents the residual variance. AM, GBLUP with additive effects only, using an additive genomic relationship; ADM, GBLUP with additive and dominance effects, using separate additive and dominance relationship matrices; ML, conventional XGBoost model using the original SNP marker matrix; ML_Transformed, XGBoost model using the transformed SNP marker matrix incorporating additive and dominance information.

Across both simulated architectures, prediction ability generally declined as PDV increased for both classical and machine learning models (**Figures 4A–C**, **5**), indicating that additive effects are captured more efficiently than dominance effects. This trend was consistent across heritability levels and trait architectures. An exception was observed for machine learning models in oligogenic scenarios, where the reduction in prediction accuracy with increasing PDV was small and not statistically significant.

Despite the overall decline in absolute prediction ability, models explicitly incorporating dominance (ADM, CADM, and ML_Transformed) generally outperformed the additive-only model (AM). The relative advantage of dominance-inclusive models increased with PDV (**Figure 5**), highlighting the growing importance of modeling dominance as its contribution to genetic variance increases.

### Performance of CADM in Capturing Dominance Effects

The locus-specific degree-of-dominance transformation was evaluated in both a classical (CADM) and a machine learning framework (ML_Transformed). The ML_Transformed model did not improve prediction performance relative to the standard ML model across simulated scenarios or heritability levels, and showed no consistent benefit in the real population data. A marginal improvement (0.6%) was observed only for the Pollen DAP trait. (as depicted in **Figures 3**–**5**).

CADM generally outperformed other classical models in both polygenic and oligogenic simulated scenarios (**Figures 4A–C**, **5**). However, this advantage did not translate to the real population, where CADM showed no consistent improvement over alternative models (**Figure 3A**).

### A genomic model outperforming machine learning

Our analysis reveals notable performance advantages of the CADM model over machine learning models in polygenic simulated scenarios, particularly evident in cases with high dominance variation (**Figure 5**). Performance improvements of up to 7% were observed when $d^{2}$ exceeded 20%, and PDV surpassed 30% (see **Supplementary Information**). However, these improvements were not consistently replicated in the data of the real population used in this study (G2F data), though low $d^{2}$ and PDV were observed. Notably, there was an exception in the case of Pollen DAP trait, where $d^{2}$ and PDV was relatively high (24% and 27%, respectively) (**Supplementary Table 3**). Nevertheless, model performance did not align with simulated population trends.

In oligogenic simulated scenarios, a completely different outcome was observed. The ML models generally outperformed the CADM model across all observed proportions of dominance variation and heritability (**Figure 5**), demonstrating GBLUP’s limitation in traits with few genes.

### ANOVA and *post hoc* significance testing

To assess the statistical significance of observed differences in predictive performance among models, we performed one-way analyses of variance (ANOVA) for each trait in the real hybrid maize dataset and simulated scenarios. Analyses were conducted to evaluate the effects of method (machine learning vs. classical) and model (AM, ADM, CADM, ML, and ML_Transformed). P-values were adjusted across traits using the Benjamini–Hochberg false discovery rate (FDR) procedure. For traits exhibiting a significant overall method or model effect (FDR < 0.05), Tukey’s honestly significant difference (HSD) *post hoc* tests were applied to identify pairwise differences between models. These analyses confirmed that differences in model and/or method performance were statistically significant (p < 0.05). Detailed ANOVA results and *post hoc* comparisons are provided in the **Supplementary Information**.

## Discussion

The ever-increasing application of genomic selection in hybrid breeding has been characterized by a constant need to improve the accuracy of genomic prediction models. Improvement has resulted in genomic prediction applications moving from the use of models incorporating additive effects only (VanRaden, 2008) to the introduction of non-additive effects incorporating models (Vitezica et al., 2013; Amadeu et al., 2020; Bajgain et al., 2020; González-Diéguez et al., 2021; Ramstein et al., 2020; Tan and Ingvarsson, 2022), and more recently, machine learning genomic models have been used in place of classical statistical models (Jeong et al., 2020; Sousa et al., 2020; Zhao et al., 2020; Montesinos López et al., 2022; Mora-Poblete et al., 2023; Sirsat et al., 2022). These recent developments in genomic modeling have been motivated by the need to maximize the contribution of non-additive effects to genomic prediction. Here, we assessed the performance of a classical model, CADM, and its ML version ML_Transformed, which incorporates a locus-specific weighted degree of dominance transformation as a prior for prediction in a hybrid maize population. We investigated how these genomic models perform under different trait architectures (different proportions of dominance variation and number of QTLs) using simulated and real hybrid maize populations (Genomes to Field Population). We found that the locus-specific weighted dominance transformation produced variable outcomes. While it did not improve the performance of ML models, it enhanced the CADM model in simulated plant breeding scenarios. Across both simulated and real maize populations, ML methods generally outperformed GBLUP, except in the polygenic simulations where CADM achieved the highest predictive accuracy. Notably, we observed a decline in model performance as the proportion of dominance variance increased, and this was regardless of the genomic method. These patterns suggest that while the transformation can be beneficial under certain genetic architectures, its utility is trait-dependent and may be most valuable for traits governed by a large number of loci with moderate to minor effects. For simplicity, this study focuses on dominance effects while omitting other non-additive effects like epistasis, which also contribute to genomic models in some cases.

### CADM model in hybrid maize

One of the primary objectives of this study was to evaluate the performance of the CADM method in a real hybrid maize breeding population. While CADM was initially proposed by Liu et al. (2022) and demonstrated superior genomic prediction ability in livestock datasets (chickens and pigs), this work represents its first application in a plant breeding population. In Liu et al.’s (2022) study, CADM outperformed classical additive and additive-dominance models, with improvements as high as 46.1% for traits like breast muscle percentage (BMP) in chickens. These improvements were evident for traits with low heritability, even when zero dominance variance was detected, suggesting that CADM may exploit dominance-related information beyond what is captured by traditional variance component estimates.

In contrast, our results in hybrid maize showed a markedly different pattern. CADM generally failed to improve predictive ability, often performing comparably or worse than classical additive and additive-dominance models, especially for traits with low dominance variance. Only one trait showed marginal improvement, Pollen DAP, which had both a high heritability and high PDV. These divergent findings suggest that the predictive benefit of CADM may be species or population-specific. These contrasting outcomes indicate that the predictive advantage of CADM observed in livestock populations may not directly translate to hybrid maize and suggest that its performance is likely contingent on species and population specific genetic features.

Several biological and population genetic factors may be the reason for the contrasting outcomes. Compared with livestock populations, maize exhibits greater genome complexity, faster LD decay, and more heterogeneous allele-frequency distributions. In addition, heterosis in maize is driven by a combination of dominance, overdominance, and epistatic interactions that may not align well with the assumptions implicit in CADM. Notably, Liu et al. (2022) proposed that CADM is particularly effective when dominance effects vary across QTLs, such heterogeneity is prevalent in hybrid maize populations as studies on maize hybrids have shown that QTLs exhibit varying dominance effects ranging from strong dominance (often partial or complete) too little to no dominance, especially for key agronomic traits like grain yield, plant height, and flowering time (Frascaroli et al., 2007; Larièpe et al., 2012; Ramstein et al., 2020). Accordingly, we anticipated comparable performance of the CADM model in the G2F hybrid maize population.

Contrary to expectations, this was not observed, suggesting that the dominance variation across QTLs did not have an effect on the underlying CADM’s improved performance reported in pig and poultry data by Liu et al. (2022). The lack of consistent performance gains suggests that variation in dominance effects across QTLs alone may be insufficient to translate into an advantage to CADM. One possible explanation is that the dominance effects in maize hybrids are highly sensitive, being strongly influenced by genetic background, allele dosage, and epistatic interactions between loci. Such effects may not be adequately captured by CADM, which extends classical genomic models but remains fundamentally linear in structure. As a result, CADM may struggle to leverage dominance information effectively when dominance effects are intertwined with complex non-linear and higher-order interactions.

Furthermore, population structure and breeding design likely played an important role. The G2F population consists of testcross hybrids derived from specific heterotic groups, and the expression of dominance effects in such populations depends critically on the degree of divergence and functional complementarity between parental lines. Previous studies have shown that the contribution of dominance to genomic prediction accuracy varies with the genetic distance between heterotic groups and the extent of within-group diversity (Gerke et al., 2015; Ramstein et al., 2020). In this context, dominance effects may be unevenly distributed across loci and environments, which can reduce their effective contribution to prediction even when modeled in a locus-specific manner, as is done in CADM.

Taken together, these findings suggest that the limited performance of CADM in hybrid maize reflects a mismatch between the model’s linear assumptions and the underlying genetic and population complexity of cross-pollinating crop species. Future studies should therefore evaluate CADM across a broader range of hybrid breeding populations, including those with greater genetic divergence, alternative crossing schemes, and multi-environment trials, to better identify and define the conditions under which CADM can provide tangible benefits. Such analyses will be essential for clarifying whether CADM’s success in livestock populations can be extended to plant breeding systems, or whether more flexible modeling is required to fully exploit dominance and non-additive effects in hybrid crops.

### ML_transformed model (a machine learning extension of CADM)

Another objective of this study was to evaluate the effect of incorporating the locus-specific dominance matrix transformation used in CADM into genomic prediction using machine learning (ML) methods in a real hybrid maize population. We selected XGBoost as our ML method of choice, primarily due to its consistently superior performance compared to other ML algorithms across diverse data science applications (Zhou and Troyanskaya, 2015; Chen and Guestrin, 2016). Its effectiveness has also been demonstrated in genomic studies (Li et al., 2018, 2019; Westhues et al., 2021; Elgart et al., 2022), where it has been shown to improve predictive ability.

Evidence gathered from our real populations suggested that, though ML models used in our studies consistently performed better than all the GBLUP-derived models (CADM inclusive), just like the results from the CADM application in the maize population, the ML_Transformed did not improve in prediction ability from the ML model, except for a single trait (Pollen DAP). This is similar to recent studies (Zhao et al., 2021; Budhlakoti et al., 2022; Mathew et al., 2022) which demonstrated to different degrees that data transformation and or modification of machine and deep learning methods can only enhance performance slightly (and in some cases lower performance) by enabling the extraction of non-linear relationships such as non-additive genetic effects (dominance or higher-order local epistatic interactions) for genomic prediction.

The poor performance of the ML_Transformed model used in this study raises several important considerations. The application of locus-specific dominance transformation in XGBoost may have been detrimental for some traits. One possibility is that the transformed features introduced redundancy or noise, disrupting the inherent feature hierarchy or interaction modeling capabilities of XGBoost, this was echoed in a study by Karwowska et al. (2025) having systematically evaluated the impact of various data transformations on binary classification performance with XGBoost, XGBoost can be susceptible to overfitting and interpretability issues when feature transformations introduce redundancy or noise, which may alter feature importance without improving overall accuracy. Another explanation is that such transformations may have interfered with XGBoost’s ability to capture complex genetic interactions, which it is otherwise well-equipped to handle through its tree structure (Friedman, 2001; Westhues et al., 2021; Fernandes et al., 2024). These findings align partially with those of (Fernandes et al., 2024), who reported similar or in some cases worse performance between models that created interactions between each hybrid in the genomic relationship matrix and each environment in the derived environmental matrix with Kronecker products, and G+E model, which only integrates genetic and environment data through the concatenation of data sets, and challenge the expectation from studies such as (Montesinos-López et al., 2024), which suggest ML models can flexibly adapt to diverse input representations. Thus, care must be taken when integrating biologically informed transformations into ML pipelines, as their utility may be model and data-dependent. Lastly, we use a large number of genomic SNP markers in this study, so there is a possibility that the additive genetic effects may have already accounted for a majority proportion of the variation, so the improvement based on dominance effects is limited (Zhao et al., 2021). Although the ML transformed model did not perform optimally in most scenarios in this study, there is considerable scope for enhancement through continued research. The transformed matrix currently factors only dominance effects. However, one viable approach for refining the ML-transformed model could involve incorporating other non-additive effects at the stage of locus-specific transformation. Another promising strategy could be to employ feature selection, a methodology known to improve the performance of non-linear algorithms (Azodi et al., 2019). Although applying feature selection could decrease the genetic variation captured, it is likely to boost the estimation of effect accuracy through the fusion of locus-specific transformation and the ML method, as Azodi et al. (2019) suggested.

### CADM & ML_transformed models in simulated hybrid maize data

The application of models that incorporate dominance in breeding and among genomic scientists is not as common as the additive effect models. However, among the cases in which dominance effect incorporating models have been used, there have been reports of significant model improvement at $d^{2}$ above 20% (Toro and Varona, 2010; de Almeida Filho et al., 2016), and in our study, similar patterns were observed. The dominance effect incorporating models used here shows a significant increase in prediction ability as $d^{2}$ gets to 20%, and the performance of these models over the base model (additive model) increases as $d^{2}$ increases. As previously reported, these results were more evident in the polygenic scenarios ( (Toro and Varona, 2010; de Almeida Filho et al., 2016). The dominance effect, incorporating models in summary, notably boosted accuracies in simulated polygenic scenarios with significant dominance effects. However, their efficacy is less pronounced when the dominance effects are minor (was also observed that there was a consistent decrease in model performance as dominance increased. This has been reported in a previous study, which was on the contribution of dominance to phenotype prediction in pine breeding and simulated populations by de Almeida Filho et al. (2016). Consequently, the decision to incorporate dominance in genomic prediction in a hybrid maize breeding scheme should hinge on the genetic architecture of the trait of interest within each population.

Our simulation results also reveal distinct performance patterns of genomic prediction models depending on the genetic architecture (polygenic vs. oligogenic), dominance variance, and broad-sense heritability (H^2^). In Polygenic Scenarios, the CADM model consistently outperformed all other models, both other classical (AM & ADM) and machine learning (ML_Transformed and ML), across all three levels of heritability. The advantage of the CADM model increased with higher dominance variance (PDV & $d^{2}$), highlighting its ability to utilize dominance variation effectively. This aligns with the application in Liu et al. (2022), who found that in animal breeding populations, transformed GBLUP improved predictive ability compared to conventional GBLUP models, especially when Heritability is high, though Liu et al. (2022) does not support the conclusion that the CADM model increases with higher dominance variance. In Oligogenic scenarios, the machine learning models demonstrated superior performance compared to the classical models, including CADM, across H^2^ levels. There are two key reasons for XGBoost’s superior performance in oligogenic scenarios compared to polygenic ones.

Firstly, XGBoost sequentially combines different predictors while applying shrinkage, a phenomenon pointed out in Friedman’s (2001) study. This variable selection process reduces the background noise in the data, allowing it to avoid diluting significant gene effects, a drawback in the GBLUP model. Secondly, similar to other non-parametric models, XGBoost does not impose strong assumptions on the phenotype-genotype relationship, thereby capturing non-linear interactions among loci, as explained by (Gianola et al., 2006; Long et al., 2010). This allows genes to be prioritized only if they contribute significantly to the model, thus reducing noise and maximizing the contribution of the non-additive effects (dominance and epistatic effects), something that would otherwise not be captured by the GBLUP model.

Across both architectures (Polygenic and oligogenic), an increase in dominance variance generally improved the relative performance of the models. However, in polygenic + high dominance variance​, the CADM model consistently dominated, suggesting a parametric dominance kernel remains the most efficient when effects are widely dispersed. In oligogenic + high dominance variance, ML retained the advantage, indicating that flexible, data-driven interaction modeling can still surpass kernel methods when the architecture is sparse. The contrasting performance of the CADM and machine learning models, although evaluated under simulated scenarios, highlights the importance of genetic architecture in predicting maize hybrid performance. CADM was more effective for polygenic traits, consistent with the assumptions of classical genomic models in which traits are governed by many small additive effects. In contrast, ML models excelled in oligogenic scenarios, likely due to their ability to capture non-linear patterns and dominance-driven effects. From a breeding perspective, these results support a trait-adaptive modeling strategy in hybrid maize breeding, whereby CADM is favored for highly polygenic traits.

Dominance incorporating genomic modeling can capture a large share of dominance variance in simulations, but real population performance is more variable. Similar to our findings in this study, which show a discrepancy between the simulated and real maize population, multiple other studies support this observation (Nishio and Satoh, 2014) found that while GBLUP, which includes the dominance genetic effect, explained over 50% of dominance genetic variance in simulation data, its performance varied significantly in real pig populations (Widener et al., 2021; Jung et al., 2022) emphasize the importance of environmental covariates and genotype-by-environment interactions in genomic prediction models, particularly for extreme environments.

Additionally, it highlights the strong phenotypic response of plants to environmental factors and underscores the need for multi-environment modeling approaches. The differences in model performance observed between simulated and real populations can be largely attributed to population-specific factors, including population structure, and genetic complexity. Simulated datasets are typically generated under controlled assumptions: allele frequencies, linkage disequilibrium, and effect sizes are often uniform, dominance effects are fixed, and environmental conditions are constant. In such idealized scenarios, models like CADM, which capture locus-specific dominance, or ML models, which can detect complex interactions, can perform close to their theoretical optimum.

In real hybrid maize populations, these assumptions no longer hold. Population structure, such as the divergence between heterotic groups, creates uneven allele frequencies and patterns of relatedness that can bias dominance effect estimates and reduce the generalizability of locus-specific models like CADM. Genetic complexity further compounds the problem: maize traits are typically polygenic, influenced by many loci with additive, dominance, and epistatic effects. Rapid LD decay and heterogeneous allele frequencies mean that locus-specific effects are more fragmented, making them harder to capture accurately, even with CADM. ML models, which can accommodate non-linear and higher-order interactions, may partially compensate for this complexity, but performance is still constrained by the underlying structure of the population. These differences in model performance between simulated and real populations show the importance of considering population-specific factors in genomic prediction. Understanding these complexities is critical for enhancing and interpreting model predictive ability and informing breeding decisions.

### Limitations

One major limitation of the transformed models is that one has to calculate a genotype matrix for each trait, which increases the computational time when working with multiple traits. This can be particularly challenging when dealing with large datasets or a high number of traits. Another is that dominance effects in genetics are not limited to complete or incomplete dominance but also include overdominance, which can vary significantly between traits. The transformed models do not account for overdominance, which may not completely match the actual situation and can lead to an incomplete representation of the genetic architecture of traits.

## Conclusions

These findings suggest that while the CADM and ML_Transformed have potential, their use may largely depend on species-specific genetic architectures. Importantly, our results reinforce the broader relevance of dominance-aware modeling in plant breeding and highlight the need for flexible, trait and species-adaptable methods in genomic prediction. Finally, together with explaining the impact of dominance variation on model performance, our findings contribute to breeding strategies optimization and genomic prediction methodologies. Further research is needed to refine these models and increase their practical implementation in breeding programs.

## Supplementary Information

A Google sheet containing all supplementary data is available at https://docs.google.com/spreadsheets/d/1rVYaQRU8hWXUsBVDmBB6hZxLwg6ss3paCxVKRU4Avlg/edit?gid=1140304086#gid=1140304086.

## Data Availability

We obtained the G2F dataset from the committee of The Genomes to Fields 2022 Maize Genotype by Environment Prediction Competition, accessible on https://doi.org/10.25739/tq5e-ak26. A GitHub repository containing the bash scripts, R scripts, and Python scripts used for the simulation analysis, phenotypic and genotypic analysis, and all the genomic predictions is available at https://github.com/brightguru/dominancetransformation-ml-paper/tree/main.

## Supplementary material

Supplementary Table 1 Variance components, narrow and broad sense heritability, and proportion of dominance variation for twelve simulated polygenic scenarios.

Supplementary Table 2 Variance components, narrow and broad sense heritability, and proportion of dominance variation for twelve simulated oligogenic scenarios.

Supplementary Table 3 Variance components, narrow and broad sense heritability, and proportion of dominance variation for seven traits of the G2F maize population.

Supplementary Table 4 Hyperparameters tuned with Bayesian optimization for XGBoost models implemented.
