# Supplementary material for: Predicting maize hybrid performance with machine learning and a locus-specific weighted degree of dominance transformation
Source: Front Plant Sci. 2026 Apr 1;17:1694707. doi: 10.3389/fpls.2026.1694707 (PMC13079612; doi:10.3389/fpls.2026.1694707)
Supplement: Supplementary Table 1 — Variance components, narrow and broad sense heritability, and proportion of dominance variation for twelve simulated polygenic scenarios. [file Table1.docx]

Supplementary Table 1: Variance components, narrow and broad sense heritability, and proportion of dominance variation for twelve simulated polygenic scenarios

|  | | Variance components | | | Heritability | |  |  |  |
| --- | --- | --- | --- | --- | --- | --- | --- | --- | --- |
| Simulated scenario | Method | Additive | Dominance | Residual | $h^{2}$ | $H^{2}$ | $d^{2}$ | PDV | $\frac{d^{2}}{h^{2}}$ |
| Poly36 | AlphaSim | 0.27 | 0.21 | 1.24 | 0.16 | 0.27 | 0.12 | 0.42 | 0.77 |
|  | ADM | 0.41 | 0.35 | 1.06 | 0.18 | 0.39 | 0.21 | 0.46 | 1.16 |
| Poly55 | AlphaSim | 0.28 | 0.22 | 0.32 | 0.35 | 0.61 | 0.27 | 0.44 | 0.77 |
|  | ADM | 0.45 | 0.25 | 0.28 | 0.33 | 0.66 | 0.33 | 0.35 | 0.99 |
| Poly45 | AlphaSim | 0.28 | 0.24 | 0.15 | 0.42 | 0.78 | 0.36 | 0.46 | 0.85 |
|  | ADM | 0.39 | 0.249 | 0.109 | 0.41 | 0.85 | 0.44 | 0.39 | 0.39 |
| Poly41 | AlphaSim | 0.40 | 0.18 | 1.42 | 0.20 | 0.29 | 0.09 | 0.31 | 0.45 |
|  | ADM | 0.48 | 0.37 | 1.22 | 0.21 | 0.39 | 0.18 | 0.43 | 0.85 |
| Poly39 | AlphaSim | 0.55 | 0.23 | 0.48 | 0.44 | 0.61 | 0.19 | 0.31 | 0.43 |
|  | ADM | 0.39 | 0.24 | 0.43 | 0.43 | 0.65 | 0.22 | 0.38 | 0.50 |
| Poly40 | AlphaSim | 0.44 | 0.21 | 0.16 | 0.58 | 0.80 | 0.27 | 0.34 | 0.47 |
|  | ADM | 0.49 | 0.19 | 0.11 | 0.55 | 0.86 | 0.31 | 0.28 | 0.56 |
| Poly62 | AlphaSim | 0.38 | 0.08 | 0.11 | 0.67 | 0.81 | 0.14 | 0.18 | 0.21 |
|  | ADM | 0.36 | 0.09 | 0.10 | 0.66 | 0.82 | 0.16 | 0.20 | 0.24 |
| Poly75 | AlphaSim | 0.34 | 0.07 | 0.91 | 0.27 | 0.30 | 0.05 | 0.17 | 0.19 |
|  | ADM | 0.37 | 0.10 | 0.89 | 0.24 | 0.31 | 0.08 | 0.21 | 0.32 |
| Poly77 | AlphaSim | 0.25 | 0.05 | 0.18 | 0.51 | 0.62 | 0.10 | 0.17 | 0.20 |
|  | ADM | 0.33 | 0.06 | 0.18 | 0.49 | 0.63 | 0.14 | 0.16 | 0.28 |
| Poly94 | AlphaSim | 0.21 | 0.00 | 0.49 | 0.30 | 0.30 | 0.00 | 0.00 | 0.00 |
|  | ADM | 0.30 | 0.00 | 0.50 | 0.29 | 0.29 | 0.00 | 0.00 | 0.00 |
| Poly91 | AlphaSim | 0.18 | 0.00 | 0.13 | 0.58 | 0.58 | 0.00 | 0.00 | 0.00 |
|  | ADM | 0.28 | 0.00 | 0.13 | 0.59 | 0.59 | 0.00 | 0.00 | 0.00 |
| Poly93 | AlphaSim | 0.32 | 0.00 | 0.09 | 0.77 | 0.77 | 0.00 | 0.00 | 0.00 |
|  | ADM | 0.31 | 0.00 | 0.08 | 0.81 | 0.81 | 0.00 | 0.00 | 0.00 |

Supplementary Table 2: Variance components, narrow and broad sense heritability, and proportion of dominance variation for twelve simulated oligogenic scenarios

|  | | Variance components | | | Heritability | |  |  |  |
| --- | --- | --- | --- | --- | --- | --- | --- | --- | --- |
| Simulated scenario | Method | Additive | Dominance | Residual | $h^{2}$ | $H^{2}$ | $d^{2}$ | PDV | $\frac{d^{2}}{h^{2}}$ |
| Oligo51 | AlphaSim | 0.37 | 0.22 | 0.15 | 0.47 | 0.81 | 0.27 | 0.34 | 0.58 |
|  | ADM | 0.38 | 0.21 | 0.16 | 0.50 | 0.79 | 0.28 | 0.36 | 0.56 |
| Oligo74 | AlphaSim | 0.25 | 0.15 | 0.25 | 0.39 | 0.60 | 0.23 | 0.38 | 0.59 |
|  | ADM | 0.23 | 0.18 | 0.23 | 0.36 | 0.64 | 0.28 | 0.43 | 0.76 |
| Oligo22 | AlphaSim | 0.33 | 0.20 | 1.12 | 0.20 | 0.31 | 0.12 | 0.38 | 0.60 |
|  | ADM | 0.32 | 0.25 | 1.10 | 0.19 | 0.34 | 0.15 | 0.43 | 0.77 |
| Oligo31 | AlphaSim | 0.41 | 0.12 | 0.12 | 0.64 | 0.81 | 0.19 | 0.23 | 0.29 |
|  | ADM | 0.50 | 0.08 | 0.17 | 0.67 | 0.77 | 0.11 | 0.14 | 0.16 |
| Oligo61 | AlphaSim | 0.31 | 0.10 | 0.28 | 0.46 | 0.58 | 0.16 | 0.27 | 0.34 |
|  | ADM | 0.38 | 0.12 | 0.26 | 0.50 | 0.66 | 0.15 | 0.23 | 0.31 |
| Oligo83 | AlphaSim | 0.48 | 0.17 | 1.35 | 0.24 | 0.32 | 0.08 | 0.27 | 0.35 |
|  | ADM | 0.45 | 0.24 | 1.30 | 0.23 | 0.35 | 0.12 | 0.35 | 0.53 |
| Oligo70 | AlphaSim | 0.60 | 0.11 | 0.17 | 0.70 | 0.80 | 0.12 | 0.16 | 0.18 |
|  | ADM | 0.59 | 0.11 | 0.17 | 0.68 | 0.81 | 0.13 | 0.16 | 0.19 |
| Oligo44 | AlphaSim | 0.25 | 0.05 | 0.19 | 0.52 | 0.61 | 0.10 | 0.17 | 0.20 |
|  | ADM | 0.27 | 0.05 | 0.19 | 0.53 | 0.62 | 0.09 | 0.15 | 0.17 |
| Oligo28 | AlphaSim | 0.62 | 0.15 | 1.72 | 0.25 | 0.31 | 0.06 | 0.20 | 0.24 |
|  | ADM | 0.84 | 0.15 | 1.79 | 0.30 | 0.36 | 0.05 | 0.15 | 0.18 |
| Oligo94 | AlphaSim | 0.52 | 0.00 | 0.13 | 0.80 | 0.80 | 0.00 | 0.00 | 0.00 |
|  | ADM | 0.27 | 0.00 | 0.13 | 0.66 | 0.68 | 0.01 | 0.02 | 0.02 |
| Oligo93 | AlphaSim | 0.27 | 0.00 | 0.19 | 0.59 | 0.59 | 0.00 | 0.00 | 0.00 |
|  | ADM | 0.31 | 0.00 | 0.18 | 0.64 | 0.64 | 0.00 | 0.00 | 0.00 |
| Oligo96 | AlphaSim | 0.22 | 0.00 | 0.52 | 0.30 | 0.30 | 0.00 | 0.00 | 0.00 |
|  | ADM | 0.27 | 0.03 | 0.48 | 0.34 | 0.38 | 0.04 | 0.10 | 0.11 |

Supplementary Table 3: Variance components, narrow and broad sense heritability, and proportion of dominance variation for seven traits of the G2F maize population

|  | | **Variance components** | | | **Heritability** | |  |  |  |
| --- | --- | --- | --- | --- | --- | --- | --- | --- | --- |
| **Traits** | Method | Additive | Dominance | Residual | $h^{2}$ | $H^{2}$ | $d^{2}$ | PDV | $\frac{d^{2}}{h^{2}}$ |
| Test weight (kg m^-3^) | ADM | 294.71 | 33.39 | 224.37 | 0.54 | 0.58 | 0.06 | 0.10 | 0.11 |
| Ear Height (cm) | ADM | 170.55 | 17.78 | 17.02 | 0.71 | 0.81 | 0.09 | 0.09 | 0.12 |
| Plant Height (cm) | ADM | 212.22 | 16.67 | 29.38 | 0.73 | 0.79 | 0.06 | 0.07 | 0.09 |
| Pollen DAP (days) | ADM | 2.58 | 0.98 | 0.52 | 0.75 | 0.92 | 0.24 | 0.27 | 0.32 |
| Silk DAP (days) | ADM | 3.20 | 0.89 | 0.52 | 0.78 | 0.92 | 0.19 | 0.22 | 0.25 |
| Grain Moisture (%) | ADM | 0.73 | 0.28 | 0.44 | 0.45 | 0.77 | 0.19 | 0.28 | 0.43 |
| Grain Yield (Mg ha⁻¹) | ADM | 0.59 | 0.08 | 0.37 | 0.44 | 0.52 | 0.07 | 0.12 | 0.17 |

Supplementary Table 4: Hyperparameters tuned with Bayesian optimization for XGBoost models implemented.

| **Hyperparameter** | **Meaning** | **Min** | **Max** |
| --- | --- | --- | --- |
| n_estimators | The number of boosting rounds or trees to be built | 3000 | 8000 |
| learning_rate | the step size at each iteration while moving towards the optimal solution. | 0.005 | 0.3 |
| max_depth | The maximum depth of a decision tree | 2 | 15 |
| min_child_weight | The minimum sum of instance weights needed in a child node | 1 | 20 |
| subsample | The fraction of training data randomly sampled for each boosting round | 0.1 | 1 |
| colsample_bytree | The fraction of features used to build each tree | 0.1 | 1 |
| reg_alpha | Adds a penalty on the absolute magnitude of feature weights | 10 | 200 |
| reg_lambda | Adds a penalty on the squared magnitude of feature weights | 1 | 10 |
| gamma | Sets the minimum loss reduction required to split a leaf node. | 0.01 | 0.5 |
